# Protective Measures for Humans against Avian Influenza A(H5N8) Outbreaks in 22 European Union/European Economic Area Countries and Israel, 2016–17

**DOI:** 10.3201/eid2410.180269

**Published:** 2018-10

**Authors:** Cornelia Adlhoch, Gavin Dabrera, Pasi Penttinen, Richard Pebody, on behalf of Country Experts

**Affiliations:** European Centre for Disease Prevention and Control, Stockholm, Sweden (C. Adlhoch, P. Penttinen);; Public Health England, London, UK (G. Dabrera, R. Pebody)

**Keywords:** influenza, avian influenza, influenza virus, viruses, H5N8 subtype, respiratory infections, birds, public health protection measures, vaccination, antiviral prophylaxis, occupational safety, zoonoses, countries, European Union, European Economic Area, Europe, Israel

## Abstract

We sought to better understand national approaches for managing potential human health risks during outbreaks of infection with avian influenza A(H5N8) virus during 2016–17. Twenty-three countries in the Union/European Economic Area and Israel participated in this study. Risk to the general public was assessed as low in 18 countries and medium in 1 country. Of 524 exposed persons identified, 274 were passively monitored and 250 were actively monitored. Of 29 persons tested, all were negative for H5N8 virus. Vaccination and antiviral drug recommendations varied across countries. A high level of personal protection was recommended although a low risk was assessed. No transmission of this virus to humans was identified.

Outbreaks of highly pathogenic avian influenza A(H5N8) pose a challenge for the food production industry and veterinary and public health services. This disease might cause losses of large numbers of animals, spread rapidly within and between countries, and entail transmission to humans. Several avian influenza outbreaks in avian species have been associated with severe illness in persons directly exposed to birds in these events (*1*–*6*). All novel influenza strains detected in humans are reportable under European Union (EU) legislation and the International Health Regulations (2005) (*7*–*9*).

Evolution, adaptation, and frequent reassortment create new avian influenza viruses that might be transmitted to humans, and determining their potential public health risk remains a challenge. Direct unprotected exposure to infected poultry (e.g., at live-bird markets or in backyard farms) is the greatest known risk for human infection (*10*). Although rapid sequencing of these viruses might identify markers for transmissibility to humans, uncertainty regarding the risk to human health might persist until sufficient data for human exposures and outcomes have been collected. International organizations have developed tools to assess such risk: these tools include the FluRisk project of the European Food Safety Authority (*11*), the Tool for Influenza Pandemic Risk Assessment of the World Health Organization (*12*), and the Influenza Risk Assessment Tool of the US Centers for Disease Control and Prevention (*13*). However, how these risks are managed locally might vary.

For early identification of new influenza viruses potentially transmissible to humans and to prevent and prepare for these threats, avian influenza surveillance is essential. The surveillance and control of avian influenza in poultry and wild birds is laid down in EU legislation (*14*,*15*). Legally mandated measures include establishing control and surveillance zones after detection of relevant viruses; these measures are limited to H5 and H7 subtypes, although other avian influenza virus subtypes have also infected humans (*16*). To control an outbreak and prevent further spread, affected poultry flocks must be quickly destroyed, which might expose the involved agricultural workers and animal health staff to avian influenza infection. This necessity poses particular challenges when avian influenza viruses differ in sequence from those already known because there will initially often be limited or no information about the risk for human illness or laboratory-confirmed infection, and exposures tend to be rare. Thus, decision makers might struggle to balance the need to control an avian influenza outbreak against the need to prevent infection among exposed persons.

After circulating in countries in Asia, H5N8 virus clade 2.3.4.4 group A was introduced into Europe during the winter of 2014–15 (*17*,*18*). Viruses of this clade had been shown to have a low ability to transmit between ferrets, to exhibit low-to-moderate virulence in mammals, and not to be transmissible by airborne infection (*19*–*21*). During and after the winter of 2016, a related, but genetically distinct influenza H5N8 virus was detected in Europe; in addition, reassortant influenza H5N5 and H5N6 viruses emerged. Because the influenza season progressed along the fall migratory routes for wild birds from Siberia across Asia into Europe, large numbers of dead wild birds and outbreaks in poultry flocks were associated with H5N8 virus clade 2.3.4.4 group B (*22*). H5N8 virus had not previously been widely seen in Europe, and the risk for bird-to-human transmission was not clear. Avian outbreaks increased in number, size, and distribution among migratory and resident birds, zoo birds, backyard farms, and poultry holdings, to a scale not previously observed for a subtype during a winter season (*23*,*24*). Overall, 24 of the 31 the European Union/European Economic Area (EU/EEA) countries, neighboring countries, Israel, and countries in the Middle East were facing large avian influenza outbreaks in poultry or wild birds during the study period, and there was great uncertainty about the risk for virus transmission from birds to humans (*25*).

Shortly after the first report of influenza H5N8 virus in Europe, the European Centre for Disease Prevention and Control (ECDC) published a risk assessment and considered the risk to the general public as being low (*26*). However, this report suggested that exposed persons should wear personal protective equipment (PPE), be followed up after exposure, and be provided with antiviral prophylaxis according to national guidelines (*26*). A first rapid follow-up assessment in 8 EU countries affected in 2014–15 that continued through December 2016 showed a heterogeneous implementation of control measures (*27*). As the outbreak continued, many more EU countries were affected, and ECDC received questions regarding optimal control measures.

The aim of this study was to describe local approaches to risk assessment and control measures used in all EU/EEA countries and Israel and to rapidly evaluate the risk for transmission to humans exposed to infected birds, to prioritize ongoing preparedness activities for future outbreaks of emerging avian influenza viruses. We hope that, as a result, countries would be enabled to harmonize their response activities related to H5N8 virus and be better prepared for future outbreaks caused by emerging avian influenza viruses.

## Methods

**Figure 1:**
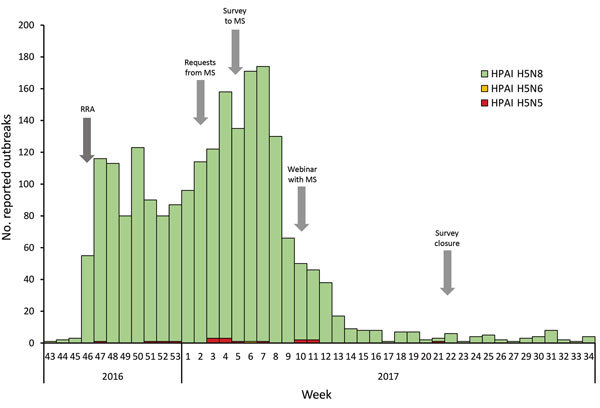
Number of reported highly pathogenic avian influenza outbreaks in birds and time line of the European Centre for Disease Prevention and Control survey (arrows), by week, European Union/European Economic Area and Israel, 2016–17. HPAI, highly pathogenic avian influenza; MS, Member State; RRA, rapid risk assessment.

We developed a structured online questionnaire to collect information on preparedness and response arrangements of each country in relation to H5N8 virus. This questionnaire covered public health risk assessments and recommendations regarding use of PPE, antiviral prophylaxis, health surveillance of exposed persons, management of symptomatic exposed persons and use of seasonal influenza vaccination, as well as cross-sector collaboration and communication. The primary design of the questionnaire was revised after discussion with several national operational contact points (OCPs) for influenza. These OCPs are nationally nominated epidemiology and virology specialists in the EU/EEA countries for any interaction with ECDC on operational matters related to influenza. On February 17, 2017, OCPs for influenza from all 31 EU/EEA Member States were invited by email to participate in this survey. Through a webinar on March 7, 2017, preliminary results were shared with the National Focal Points and OCPs. In addition, Israel contacted ECDC to inquire about protection measures during H5N8 outbreaks in affected EU/EEA countries and was also invited to participate in this study. The online survey was closed on May 31, 2017 (Figure 1).

The total number of countries invited (31 EU/EEA plus Israel) was used to calculate the response rate. We analyzed responses by using simple descriptive statistics with the number of respondents as the denominator, including numbers of countries with risk assessments at the national and individual-outbreak levels, content of national recommendations for exposed persons, provision of antiviral prophylaxis, and use of seasonal influenza vaccination. We also summarized the types of case definitions used for symptomatic possible human cases of avian influenza among exposed persons, as well as the number of persons exposed and tested for possible avian influenza infection.

## Results

**Figure 2:**
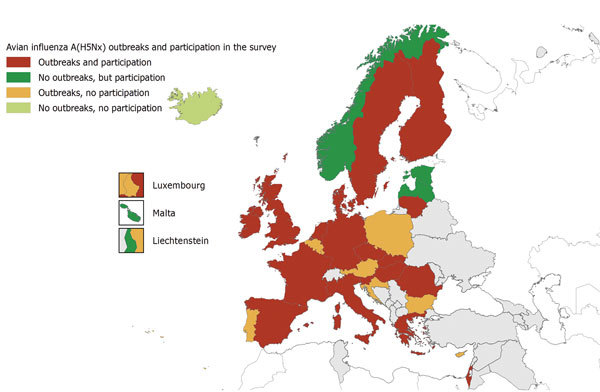
Reported highly pathogenic avian influenza outbreaks and participation in the European Centre for Disease Prevention and Control survey, by country, European Union/European Economic Area and Israel, 2016–17.

Of the 32 countries invited to complete the questionnaire, 23 (72%) responded: 22 EU/EEA Member States and Israel. Of these 23 countries, 18 reported avian influenza outbreaks; 5 did not report any outbreaks but provided a comprehensive picture of their preparedness and response (Figure 2).

National risk assessments in relation to H5N8 virus were conducted in 19 (83%) of 23 countries, and all but 2 assessed the public health risk as low, negligible, or very low. For the remaining 2 countries, 1 reported a medium risk to public health and 1 reported a low risk for the general public but a real risk for poultry workers. The real risk was based on the assumption that single infections in humans after direct contact with sick birds cannot be excluded. In 1 country, the risk assessment was performed together with veterinary experts and also updated over the course of the outbreaks. Twenty-two countries reported that their assessment considered the rapid risk assessment of ECDC. One country referred to its past experience with outbreaks of H5N8 influenza during 2014–15, and 7 referred to the fact that, to date, no infections in humans had been observed worldwide. Thirteen (59%) of 22 countries reported that local risk assessments were also undertaken in relation to individual poultry outbreaks or detection of H5N8 virus in wild birds.

### Recommendations for Protection of Persons Exposed to H5N8 Virus

Guidelines for the most relevant occupational groups responding to detections of H5N8 virus were in place in each participating country; >85% of countries had recommendations for farmers (20 countries), cullers (20 countries), and veterinarians (22 countries). Seventeen countries (81%) had recommendations for members of the public directly exposed to infected birds. Six countries had recommendations for specific exposed groups, such as firefighters, police officers, and rescuers; employees involved in culling; fishery inspectors; hunters; taxidermists; bird ringers; ornithologists; and birdwatchers.

Most guidelines for PPE recommended during wild bird outbreaks included use of gloves (21/23 countries), goggles (19/23), masks (19/23), and body suits (18/23). In addition, boots (3 countries), disinfection material (1), disposable aprons (1), handwashing (1), disposable hair nets (1), disposable gloves (1), and minimum filtering facepiece 2 (FFP2) masks (1) were recommended.

Recommended PPE to be used during poultry outbreaks included goggles (21/22 countries), gloves (21/22), masks (21/22), and body suits (21/23). Other materials and measures required in some countries were boots/rubber boots (6 countries), FFP3 respirator or mask (2), visor (1), protective gloves (1), disposable aprons (1), and covering of the hair (1).

For all but 4 countries, PPE recommendations were the same for influenza outbreaks in poultry and wild birds. In Italy, Israel, Norway, and Sweden, recommendations for detection of influenza outbreaks in wild birds were either less stringent than those for outbreaks in poultry or not documented at the national level. For example, in Norway, differences were related to the type of mask (minimum FFP2 for outbreaks in wild birds, protection level 3 for outbreaks in poultry) and type of gloves (disposable gloves for outbreaks in wild birds and thicker protection gloves for outbreaks in poultry).

### Identification and Follow-Up of Exposed Persons

In 10 countries, exposed persons were identified through the local veterinary authority alone or jointly by public health authorities together with local veterinary authorities, food safety authorities, or in liaison with the Department of Agriculture and the Marine. Other ways of identification included epidemiologic investigations or self-monitoring and reporting by the local practitioner to the regional public health authority.

Thirteen countries reported definitions of different levels of exposure that varied in their level of detail (Table 1). Major categories included type of protection (protected, unprotected, not wearing appropriate PPE, PPE breach); type of contact (indirect, direct [<1 m], skin contact, aerosol); duration of contact (accidentally short, sporadic, over longer period); type of contamination (environment, bird/dropping, birds in backyard holding area, poultry on farm); context of exposure (occupational, nonoccupational); type of setting for the outbreak (wild bird, backyard, poultry farm); type of activity (wild bird ringing, swabbing, culling, disposal, clean-up); and confirmation status of avian influenza virus (suspected, confirmed, status unclear, status cannot be confirmed).

**Table 1:** Levels of exposure, groups for active and passive follow up and recommendations for antiviral prophylaxis, by country, European Union/European Economic Area and Israel, 2016–17*

Country	Definition of level of exposure	Groups for active follow up	Groups for passive follow up	Antiviral treatment recommendations and other measures
Czech Republic	None	None	Cullers	People in close contact with infected birds and experiencing relevant health problems are advised by public health authorities to seek medical care. General practitioners/clinicians are responsible for the next therapeutic and prophylactic steps.
Denmark	Sporadic contact with birds and their droppings; workers who collect dead wild birds suspected for avian influenza; farmers, veterinarians and workers involved in handling of outbreaks with confirmed highly pathogenic avian influenza in poultry	None	Groups 2 and 3	Antiviral prophylaxis is only recommended for persons exposed at risk level 3
Estonia	None	In accordance with occupational risk analysis results	In accordance with occupational risk analysis results	For exposed persons, mostly for occupationally exposed persons
Finland	Depending on type of exposure	Exposed humans without PPE and those taking part in culling and cleaning	Others	For severe cases and risk groups, outbreak control
France	None	None; started active surveillance in 2015 but was rapidly overloaded and stopped	Exposed persons	No prophylaxis
Germany (*28*)	A) Persons in direct contact to animals possibly infected with A/H5 virus (mainly poultry workers and veterinarians); increasing risk for exposure related to duty. B) Persons in direct contact with persons possibly infected with A/H5 virus. B1) Family members or persons in the same household with a probable or confirmed case if infection with A/H5 virus. B2) Medical personnel in practices and hospitals with <1 possible, probable, or confirmed human case of infection with A/H5 virus. C) Personnel in laboratories analyzing samples suspected to contain A/H5 viruses	It is the responsibility of local health authorities to decide on the measures taken; RKI recommends using a monitoring instrument (*29*)		The decision to use antiviral drugs s up to the respective Federal State. RKI recommends to offer antiviral postexposure prophylaxis. Recommended measures for groups: A) 1. Minimize number exposed, 2. Use personal protective clothing, 3. Use measures when leaving the site, 4. Antiviral prophylaxis, 5. Vaccination; B1) 1. Antiviral prophylaxis, 2. Active follow up of contact persons, 3. Investigation and differential diagnostics of acute respiratory symptoms; B2) 1. Hygiene, 2. Antiviral prophylaxis, 3. Active follow up of contact persons, 4. Investigation and differential diagnostics of acute respiratory symptoms; C) Work performed under safety cabinet level 2 for diagnostic work; virus culture under BSL-3 conditions
Greece	Setting (exposure to wild birds/domestic poultry/occupationally exposed); type of exposure	All persons in contact with birds with high suspicion of or confirmed influenza A(H5N8( virus infection are monitored by public health professionals from the local prefecture	Local veterinary services should provide a list of those exposed to the local public health officials who are involved in active surveillance	As a general recommendation, antiviral prophylaxis is offered to persons exposed to infected birds. A risk assessment is conducted for each incident by local public health authorities and the Hellenic Center for Disease Control and Prevention, including the need for antiviral drugs. If relevant clinical symptoms develop in a person within the 10 d follow-up period, after exposure to infected birds, he or she should be given antiviral drugs at treatment dose for 5 d. Other measures include minimize number of persons exposed, use of full PPE, and vaccination
Hungary	None	Persons exposed to animals with confirmed avian influenza virus, such as influenza A(H5N8) virus	None	Exposed persons should be provided oseltamivir antiviral prophylaxis for 10 d
Ireland	Category A: Occupational persons who are exposed to avian influenza before identification of an incident who were not wearing appropriate PPE at all times during exposure. This category could include farm workers, other exposed workers, owners of backyard flocks or other persons resident at the premises who have had exposure to birds or infected materials and veterinary staff. Category B: Persons who will be occupationally exposed during the response to the incident, while wearing appropriate PPE. This category could include anyone involved in culling, disposal, and clean-up operations at a premises or rendering facilities or rangers/veterinarians capturing wild birds. Category C: Nonoccupational exposures: might include members of the public (or others) inadvertently handling sick or dead birds, or their fecal matter that is confirmed to be infected with avian influenza virus. These persons are unlikely to have been using appropriate PPE. Category D: Members of the public or others outside of occupational settings, inadvertently handling sick or dead birds, or their fecal matter for which avian influenza status cannot be confirmed (e.g., single or large bird die-off). These persons will generally be managed under the standard approach, unless information or risk assessment suggests a different approach.	Depending on risk assessment, a strict or standard approach is undertaken with regard to active surveillance. **Strict Approach**, Category A: Active follow up is required for every day up to 10 d from the last date when exposure occurred without complete PPE. This active follow up consists of daily contact between healthcare personnel and the person to check whether symptoms compatible with human avian influenza (including conjunctivitis) have developed in the person. The person should also receive standard information on potential symptoms and emergency contact instructions for healthcare personnel (for instances when symptoms develop between daily contacts). If PPE were started at a later date after an unprotected exposure, then the contact should be reassigned to passive follow up after the end of the active follow up period. Passive follow up should be continued for 10 d after the last exposure. Category B: If the person has been exposed to the incident while wearing complete PPE during all exposures, the person should undergo passive follow up until 10 d after the last exposure to the infected site. Passive follow up involves provision of information on human avian influenza symptoms for persons to be aware of and emergency contact instructions for healthcare personnel. Any person who has not worn complete PPE during all exposures will require active follow up according to Category A from the date of the last exposure without full PPE. In situations in which a person has unprotected exposure, followed by protected exposure with complete PPE, then the person should have 10 d of active follow up from the date of last exposure without complete PPE. The person should then be given instructions for passive follow up for a period <10 d after the last exposure with complete PPE. Category C: To be considered for active follow up for 10 d from the date of exposure. Category D: Not usually applicable to the strict approach. **Standard Approach,** Categories A–C: All persons exposed to infected site or birds should undergo passive follow up for 10 d after the last exposure. Category D: On the basis of risk assessment by public health or occupational health personnel; possible approaches might include consideration of passive follow up for a 1 bird without avian influenza confirmation. Active follow up might be considered for a large bird die-off in which avian influenza has not been confirmed.		Incidents regarding poultry: the decision of using chemoprophylaxis is dependent on whether a strict or standard approach is deemed appropriate. **Chemoprophylaxis:** Chemoprophylaxis should be started <7 d after the last exposure, the dose for oseltamivir is 75 mg. 1×/day. The minimum course for oseltamivir is usually for 10 d. For influenza A(H7N9), 75 mg of oseltamivir, 2×/d, is recommended for prophylaxis. Because of concerns over potential resistance to oseltamivir. If any persons are unable to take oseltamivir, this situation should be discussed with the NVRL. **Strict approach (categories A–C**). For all avian influenza incidents considered to require a strict approach, antiviral chemoprophylaxis is advised. This decision is likely to include all incidents in which the subtype of avian influenza virus is H5, H7, or H9. However, chemoprophylaxis of responders to incidents should be considered on a case-by-case basis, taking into account the evidence of the ability of the virus to cause human infection or severe disease. If in doubt, discuss with the NVRL and HPSC. The exception to the recommendations above would be for persons in Category B (wearing full PPE) who have responded to a wild bird incident, which requires a risk assessment on an individual incident basis. For only occupational contacts, prophylaxis should be started before persons have contact with birds and should be given daily while in contact and for 10 d after last exposure. If exposure has already occurred, prophylaxis should be started <7 days of the last exposure. The maximum recommended duration of prophylaxis is 42 d, and advice should be sought if it is likely to be required for longer than this period. **Standard approach (all categories):** Antiviral chemoprophylaxis is not routinely advised as long as all onditions for use of the Standard Approach are met. The standard and strict approaches were outlined in previous questions.
Israel	Protected and unprotected exposure	Unprotected exposure	Protected workers	Not recommended
Italy	None	None. Potentially exposed persons are requested to seek care by a general practitioner in case of ILI/ARI/conjunctivitis onset. In case of suspected and confirmed human ILI or ARI cases, active surveillance for close contacts is immediately activated for >10 d	All persons exposed (farmers, veterinarians, cullers)	Treatment and prophylaxis during influenza A(H5N8) outbreaks
Liechtenstein	Professional contact with poultry or wild birds; accidental contact with poultry or wild birds	None	Diagnosis of avian influenza must be reported to the healthcare system regardless of outbreak situation or group	Usual hygienic measures. No specific antiviral medication
Malta	None	All those whose work involves direct close contact with live or dead poultry/wild birds	Healthcare workers taking care of confirmed case-patients who wore PPE, and family members/friends/work colleagues who had close contact with exposed persons	For treatment of suspected and confirmed cases of avian influenza
Netherlands	1) Persons with prolonged exposure to infected animals (farmers and family, workers on the farm); 2) Persons with short period of intensive exposure to infected animals or products of infected animals (veterinarians, cullers); 3) Persons with one-time or short presence on infected farm without direct exposure to infected animals or their products; 4) Municipal Health Service personnel involved in taking specimens from patients suspected for infection with avian influenza virus.	None	Groups 1 and 2	Restrictive antiviral treatment depending on the level of exposure prophylaxis is offered alone or combined with seasonal Influenza vaccine or monitoring
Norway	1) Sporadic contact with wild birds and their droppings; 2) Close contact with sick or dead wild birds where avian influenza infection is suspected; 3) Close contact with poultry holdings where avian influenza infection is suspected or confirmed.	None	The municipality doctor is responsible for logging the name and address of exposed persons and the period of exposure. Self-monitoring for ILI, ARI, conjunctivitis, or general signs of infection should be performed for 10 d postexposure by persons at risk level 3. If symptomatic, they should contact their general practitioner and inform him or her about the exposure.	In general, antiviral prophylaxis is not recommended. Antiviral drugs are only used for influenza patients, in particular those who have an increased risk for severe illness. Neuraminidase inhibitors should be given as soon as possible and <48 h of symptom onset. Treatment for hospitalized patients should always be considered, also after 48 h of symptom onset. For avian influenza, oseltamivir is recommended for all persons at risk level 3 from first exposure to >7 d postexposure. This treatment should also be considered for persons at risk level 2.
Romania	None	Exposed persons with occupational risk and exposed persons living in/near the households where the outbreak was identified.	No particular group (general population)	Antiviral prophylaxis is not recommended. Antiviral drugs are used only for treatment when needed.
Slovak Republic	Direct contact to poultry with influenza A(H5N8) confirmation; contact with wild birds with influenza A(H5N8) confirmation; stay in environment with influenza A(H5N8) detection; contact with a person with confirmed influenza A(H5N8)	Farmer, culler, veterinarian, public directly exposed to birds	None	Public health recommendations on antiviral prophylaxis as per ECDC rapid risk assessment
Slovenia	Accidental contact with dead or diseased wild bird; professional contact with dead or diseased wild bird; professional contact with diseased poultry (no cases so far in this country)	Only for those who had intensive unprotected contact with poultry that had confirmed avian influenza infection	There is no passive surveillance in place. Nevertheless, National Influenza Centre would test any specimen positive for influenza A virus but negative for seasonal H1 or H3 subtype avian influenza viruses	Antiviral prophylaxis has not been recommended for influenza A(H5N8) and A(H5N5), but for unprotected close contact with wild birds or poultry with confirmed HPAI A(H5N1), antiviral prophylaxis would be given to exposed persons
Spain	None	None	If exposed persons have symptoms <7 d after the last contact with infected birds, they should inform public health authorities	Generally not recommended
Sweden	Exposed without protective equipment	None	For influenza A(H5N8); only passive surveillance	Not relevant for influenza A(H5N8). Antiviral treatment only for confirmed cases or during the time it takes for a diagnosis if a person has severe illness.
United Kingdom	Highest risk: culling, swabbing, direct contact with carcasses, fecal materials; other exposures are also considered to be at lower risk but still require PPE to be used.	All exposed persons (not just highest risk). However, previous recommendations had active surveillance only for those without both PPE and antiviral during exposures.	Not applicable for current recommendations. Previously for those had used PPE and antiviral drugs during all exposures.	In February 2016, interim recommendations were adopted whereby only persons with the highest risk exposures (culling, handling carcasses, direct contact with infected materials, swabbing) and had a breach in the recommended PPE required postexposure antiviral prophylaxis for 10 d.

*ARI, acute respiratory infection; BSL-3, Biosafety Level 3; ECDC, European Centre for Disease Prevention and Control; HPAI, highly pathogenic avian influenza; HPSC, Health Protection Scotland; ILI, influenza-like illness; NVRL, national virus reference laboratory; PPE, personal protective equipment; RKI, Robert Koch Institute.

All but 1 of the participating countries undertook surveillance of exposed persons according to national guidelines. Ten countries reported active follow-up (i.e., exposed persons were proactively contacted to check on their health status). Thirteen countries reported passive follow-up (i.e., exposed persons were given health advice and instructions on what to do when symptomatic). In 1 country, the decision on whom to follow up and in which way was taken at the local level. Five countries considered active monitoring for all persons in close and direct contact with infected birds, and 5 other countries considered active surveillance only for persons who were unprotected when exposed. In the remaining countries, no active monitoring was undertaken. One country replied that because of a heavy workload, active surveillance activities had to be stopped. One country had to follow up persons across national borders within the EU.

Eighteen countries used case definitions for acute respiratory infection or influenza-like illness (i.e., fever with any symptoms of acute respiratory infection) in association with exposure to an infected flock/bird as a case definition to identify a possible human case of avian influenza: 10 countries used both syndromes, and 4 countries used either acute respiratory infection or influenza-like illness. Six countries used conjunctivitis, 1 country included severe acute respiratory infection, and another country used gastrointestinal symptoms in association with exposure.

Six countries reported to have actively followed up 254 exposed persons, and 4 countries reported to have passively followed up 274 persons. Five countries did not have this information at the national level, and 7 countries reported that none of any exposed persons had been monitored. Six countries reported that they had tested 29 persons for H5N8 virus; all test results were negative, but 1 person was positive for seasonal H3N2 virus.

The Netherlands and the United Kingdom reported planning to evaluate the transmission risk for exposed persons. The Netherlands reported conducting a serosurvey to follow up exposed persons and identify any possible transmission events.

### Antiviral Prophylaxis and Vaccination

Eight countries reported that antiviral prophylaxis was either not relevant for H5N8 or not recommended. The remaining countries reported that use of antiviral drugs for exposed persons differed depending on level of protection and exposure according to specific recommendations. During outbreaks, antiviral drugs were more commonly provided when exposed persons had not been wearing appropriate PPE or a breach of PPE had been reported (Table 2). Other exposed persons without PPE who would be offered postexposure prophylaxis included family members of poultry farmers, members of the public handling sick or dead birds or their fecal matter, contact persons of confirmed case-patients infected with avian influenza, and occupationally exposed persons wearing PPE but in whom influenza-like symptoms developed.

**Table 2:** Recommendation for antivirus prophylaxis for different exposed groups, European Union/European Economic Area and Israel, 2016–17*

Antivirus treatment	No. countries with recommendations			
	Occupationally exposed persons wearing PPE, n = 21	Occupationally exposed persons not wearing PPE or PPE breach, n = 22	Occupational groups handling sick or dead poultry and birds, n = 21	Other exposed persons, n = 21
No prophylaxis	14	11	13	12
Preexposure prophylaxis	2	2	2	1
Preexposure and postexposure prophylaxis	1	1	1	1
Postexposure prophylaxis	3	8	2	4
Unknown or no answer	1	0	3	3

*PPE, personal protective equipment.

Most (15/22, 68%) countries reported a recommendation for seasonal influenza vaccination of poultry workers in general and a similar (13/21, 62%) number reported to have actually recommended vaccination during these outbreaks. During the webinar, participants explained that the period between vaccination and involvement in culling operations was assessed as being too short for sufficient protection at the individual level.

### Cross-Collaboration and Communication

Most (16/21, 76%) respondents reported that animal and public health laboratories cooperated by sharing specimens. Two thirds of respondents also reported that information had been shared with primary care providers (14/21, 67%) and local hospitals (14/20, 70%) to increase awareness of local avian influenza outbreaks.

## Discussion

During the winter of 2016–17, Europe faced the longest period of avian influenza outbreaks ever recorded. These outbreaks required ≈25 million poultry to be destroyed or culled as part of preventive action (*30*). According to the EU reference laboratory for avian influenza, as October 4, 2017, EU Member States had reported 2,781 outbreaks of H5N8 influenza in poultry, wild birds, and captive birds, as well as 20 outbreaks of H5N5 influenza and 1 outbreak of H5N6 influenza in a wild bird (*31*). In our study, 18 of the 25 avian influenza–affected countries provided feedback, and 5 countries not affected also participated.

After initial identification of a new avian influenza reassortant virus, there was uncertainty about transmissibility of the virus to humans. The large number of culled birds provides numerous possible exposure events for humans and also provides useful opportunities to understand potential transmissibility of this virus from birds to humans. No laboratory-confirmed cases among symptomatic persons have been identified, although a large number of workers were involved.

Despite large numbers of workers exposed to infected birds across the affected countries, of the 195 exposed workers who were identified exhibiting symptoms of influenza-like illness or acute respiratory infection and were laboratory tested, all showed negative results. This finding could be caused by strict prevention and control measures applied in combination with an assumed poor ability of the virus to be transmitted to humans (as with other avian influenza viruses) or a combination of both factors. However, not all persons involved during outbreaks and exposed to infected birds were identified and followed up, resulting in a still high level of uncertainty. Also, asymptomatic transmission would not have been detected. Follow-up activities are resource intensive, and 1 country, for example, had to stop active follow-up of exposed persons because of limited capacity, and more risk-based passive approaches had to be implemented. Such an adapted risk-based approach for the management of exposed persons could help prioritize use of sparse resources in the most efficient way and would be especially useful if avian influenza viruses that are more transmissible from birds to humans were introduced into Europe or were to emerge.

Recommendations for the use of antiviral prophylaxis also differed among the countries. The number of countries recommending antiviral drugs increased from 30% to 50% when unprotected exposure to an infected bird was observed. However, it is unknown how well the recommendations were followed onsite. It is similarly not possible to comment on the extent to which antiviral prophylaxis for exposed persons contributed to the observed absence of acute infections with H5N8 virus despite following up >500 persons. As commented previously, asymptomatic infections could have occurred and would not have been identified by the follow-up processes described in this study.

During these outbreaks, many countries had to rely on different authorities for follow-up of exposed persons. Different responsibilities between these authorities and local, regional, and national levels hampered comprehensive information about numbers of persons exposed. Only 9 countries were able to provide at least some veterinary data, which suggested that ≈1,570 persons were exposed during 258 outbreaks (*30*). The total number of exposed persons or exposure events during outbreaks in all affected countries remains unknown. If an avian influenza virus with the capacity to be transmitted to humans enters Europe or emerges, it will be necessary to have comprehensive systems able to better identify and follow up exposed persons, test suspected cases, recognize severe diseases and prevent spread to the community.

Most avian influenza outbreaks occurred during the same time as seasonal influenza outbreaks when H3N2 viruses were dominant across Europe and caused high disease burden and excess mortality rates in many EU/EEA countries (*32*). Detection of seasonal influenza viruses in persons also exposed to avian influenza viruses highlights the possibility and risk for reassortment events. Therefore, vaccination with seasonal influenza vaccine for occupationally exposed persons needs to be carefully reassessed whenever facing avian influenza outbreaks. Considerations should include whether these outbreaks coincide with circulation of seasonal influenza viruses and whether seasonal influenza vaccine is available in sufficient quantities.

The avian influenza outbreaks described in this report show examples of successful cooperation between veterinary and public health services in some countries in sharing specimens and collaboration to identify exposed persons. However, challenges remain and efforts toward a One Health approach should be continued. Serosurveys in epidemiologic studies to follow up exposed cohorts and identify possible transmission events might better describe the risk for transmission and help reduce uncertainty when assessing the risk for those newly emerging viruses, despite known limitations regarding subtype specificity of serologic testing (*33**,**34*). Despite a wide diversity in the recommendations for use of PPE and of antiviral prophylaxis for exposed persons, we found no evidence of bird-to-human transmission of infection. The reasons for this finding might be related to the need for a minimum level of PPE and use of antiviral drugs, although another useful potential factor is likely to be the poor ability of the virus to be transmitted to humans. Finally, a thorough review of the measures recommended during these outbreaks in view of the results of our survey might help to identify critical points and challenges in each country that can be addressed and resolved to be better prepared for future outbreaks (*35*).

In conclusion, this study enabled us to put preparedness and control activities during an avian influenza emergency into a realistic context. The information gathered provided a clearer understanding of measures taken also as examples for countries not affected at the time of the survey.
